# Awareness of consanguineous marriage burden and willingness towards premarital genetic testing in Sudan: a national cross-sectional study

**DOI:** 10.1097/MS9.0000000000002210

**Published:** 2024-05-28

**Authors:** Fatima M. Elmugadam, Haythem Ahmed, MOHAMMED KARAMELGHANI, Almigdad Ali, Israa Ali, Almegdad Ahmed, Mohammed Salman, Wadah Mohamed, Elhami A. Ahmed, Khabab Abbasher Hussien Mohamed Ahmed, Ghassan E. Mustafa Ahmed, Liena Elsayed, Ahmed Musa

**Affiliations:** a Institute of Endemic Diseases; b Faculty of Medicine; c UNESCO Chair in Bioethics; d Institute of Endemic Diseases, University of Khartoum, Khartoum, Sudan; e Department of Basic Sciences, Princess Nourah bint Abdulrahman University, Riyadh, Saudi Arabia

**Keywords:** community awareness, consanguineous marriage, genetic diseases, genetic testing, Sudan

## Abstract

**Background:**

Despite the widespread practice of consanguinity in Sudan, there is a lack of exploration into the community’s awareness of its health implications on offspring and their overall attitude towards consanguineous unions.

**Aim:**

This study aimed to evaluate the community’s awareness of the possible health adversities of consanguinity on children and assess the effect of knowledge level on the prevailing attitude towards this practice in Sudan.

**Methods:**

From August to December 2018, data were collected from adults aged 18 years and above in five provinces of Sudan regardless of their marital status. The analysis involved both descriptive and multivariate statistical techniques.

**Results:**

This study revealed a consanguinity rate of 30.2%. Despite a high awareness level (73.7%) regarding the effects of consanguineous marriage on the health of the offspring, a moderately negative attitude towards this practice (63.9%) was observed.

**Conclusion:**

The discordance between the high consanguinity rate in the Sudanese population and the moderately negative attitude suggests a potential persistence of this practice in the future. Without the implementation of educational programs and the provision of genetic counselling services to consanguineous couples, the prevalence of consanguinity is likely to endure.

## Introduction

Highlights

- Although consanguinity is a common practice in Sudan, little research has been done on the community’s understanding of the health risks to kids and attitudes towards consanguineous marriages in general.
- The purpose of this study was to determine the community’s understanding of the potential health risks that consanguinity may pose to children as well as the impact that information has on the general perception of this practice in Sudan.
- The discrepancy between the Sudanese population’s high consanguinity rate and their generally unfavourable attitudes points to the possibility that this practice may continue in the future.
- The predominance of consanguinity is likely to persist if educational initiatives are not put in place and consanguineous spouses are not offered genetic counselling services.

Consanguinity or Inbreeding are the terms commonly used to describe unions between individuals who share at least one common ancestor^1^. In clinical genetics, consanguineous marriages (CM) are defined as the mating of a couple who are related as second cousins or closer^2^, although, consanguineous unions beyond second cousins do exist. The long and excessive practice of inbreeding negatively affects the overall genetics of a population, causing what is known as inbreeding depression^2–4^, but there is a specific association between consanguinity and autosomal recessive disorders^2^. In terms of mathematics, consanguinity increases the chance of mating between two individual heterozygotes for the same recessive mutant allele, causing hidden diseases in the family to appear in the next generations^1,5^.

It is globally estimated that over 20% of different communities prefer intra-familial marriages, and over 8.5% of all children in the world are the outcome of such unions^1,6,7^. However, the rates of consanguineous marriage vary worldwide. Low prevalence is found in Western countries with a 1–10% range in regions such as North America, Australia and most of Europe, the Iberian Peninsula, Japan, and South America^2^. On the contrary, CM is widely practiced in some countries in Asia, Africa, and the Arab world^1^. While the MENA region records some of the highest consanguinity rates from 20 to 50%^3,8^.

Included in the MENA region, Sudan was found to have a consanguinity rate as high as 44–63%^8^. Although there is a lack of updated national statistics, in 1995, the Federal Ministry of Health reported that 65% of women in Sudan were married to an extended family member^9^. Wherein a study in Khartoum Hospital, Sudan, reported that 49.5% of women were married to a first cousin, and 14% married to more distant relatives^10^.

There are many socio-economic and psycho-social factors contributing to the continuation of this practice, despite possible adversities^3,7^. While Intra-familial marriage is considered taboo, and endogamy is a prohibited practice in some communities, it is permissible in others such as Jewish communities, and is not advised for but allowed in Islam^11^. In Sudan, a majority of Muslim country, first-cousin unions are the preferred union pattern constituting about half of all consanguineous marriages^12^. While the rate of second-cousin marriage and more distant consanguinity is lesser, and no uncle-niece or aunt-nephew marriage was ever recorded^12^. The high inbreeding level in Sudan could be promoted by the geographical isolation of the great variety of ethnic and linguistic groups of a large number of tribes in Sudan^12–14^. No published data were investigated the socio-cultural factors of CM in the Sudanese population, but one inbreeding study has noticed the contribution of the extended family system in the continuation of this practice^10^. However, the issues of genetics and heredity have come recently to grasp attention in Sudan.

The issue of consanguinity is complex, although it is practiced for certain social benefits, it can also form a measure of health hazard in closed populations. In Genetics, the degree of consanguinity is measured using an “inbreeding co-efficient”, which implies that a greater percentage of the homozygous genome will be shared between blood relatives^7,15^. This effect is directly related to the degree of consanguinity, where the closer the relationship, the greater it is expected^5,15,16^.

Sudan is a developing country where genetic medicine has been overshadowed by other diseases regarded as the common causes of morbidity and mortality^17^. However, there have been studies commenced on the genetics of the Sudanese population, where homozygosity and inbreeding coefficients were investigated in several Sudanese tribes^18–20^. Genetic diseases augmented by CM were also studied, such as hereditary spastic paraplegia in neuro-genetics^21^, and sickle cell disease in haematological disorders^22–24^. Others like infectious diseases, metabolic diseases, psychiatric illnesses, and cancer were also studied on a molecular background^20,25–27^.

In populations with high consanguinity rates, community programs for premarital screening to detect carriers are important^16^. Genetic testing can be provided at different stages doe preventive or diagnostic measures, such as predictive tests in premarital genetic testing, prenatal and preimplantation genetic tests; or screening tests for early diagnosis like in new-born screening, or the screening for undetected cases at the level of population, and diagnostic molecular tests^28^. However, the WHO and governmental health bodies have recommended community genetics programs be established in low- and middle-income countries (LMICs, as defined by the World Bank)^3^. The WHO has also recommended an approach to minimize the negative effects of consanguinity on child health via the identification of families with a high risk of a genetic disease and the provision of prospective genetic counselling and testing^29^.

This has necessitated our study to investigate the Sudanese population on the issue of consanguinity. We look at our population as future consumers of genetic services, including community-based awareness programs concerned with the issues of heredity and health.

## Methodology

### Study design

This was a national cross-sectional community-based study.

### Data and variables

The study was conducted between August and December 2018, employing an interview-based data-capture approach following a non-randomized methodology. Our interviewers underwent training on a three-section questionnaire designed to assess the knowledge, attitude, and practice components of the subject under investigation. The questionnaire was piloted in Khartoum, central province to ensure the suitability questions to the average person’s understanding. The research spanned 13 distinct areas located in five different provinces in Sudan, each characterized by varying socio-cultural status.

Within the targeted areas, we conducted interviews with individuals from surrounding villages who frequented central marketplaces. The central province served as a representative pool for the diverse ethnicities comprising the Sudanese population. Consequently, a majority of our respondents (459 out of 1089) were interviewed in different localities within this province. The remaining respondents were systematically distributed among the four other provinces to ensure comprehensive coverage of the major ethnic and tribal compositions across the Sudanese population.

### Statistical analysis

The data were analyzed using the SPSS statistical package (version 23) and Excel sheets (program version 2018). Both descriptive and inferential statistical techniques were employed in the analysis. Descriptive analyses were conducted to outline the general characteristics of the sample. Bivariate analysis was undertaken to explore the inter-relationship between an outcome variable and an explanatory variable. In the data analysis using SPSS, dichotomous variables, representing binary choices, were typically coded with 0 and 1, corresponding to options like “No” and “Yes.” Ordinal variables, encompassing more than two categories, received numerical assignments (e.g. 1, 2, 3, 4 etc.). Missing values were always coded as (0).

The outcome variables included consanguinity, attitude towards consanguineous marriage, and awareness of the congenital effects of consanguinity. Explanatory variables encompassed demographic and socio-economic factors. The statistical significance of the bivariate relationship was assessed using a chi-square (χ²) test, given that all variables considered in this study were categorical. A *p* value less than 0.05 was deemed significant.

To identify adjusted significant predictors of attitude towards consanguinity and premarital genetic testing, a multivariate logistic regression model was applied. In this model, the attitude towards consanguinity and premarital genetic testing were considered as dichotomous dependent variables, while socio-economic, demographic, and behavioural factors served as explanatory variables.

The work has been reported in accordance with STROCSS guidelines^30^.

## Results

### Demographic characteristics of the study participants

A total of 1089 participants were involved in the study, 52.4% (571/1089) of them were females, while 47.6% (518/1089) were males. Regarding the age of the participants, 62.2% (677/1089) were aged between 19 and 35 years, while 28.5% (310/1089) were aged between 36 and 55 years. There were 7.2% (78/1089) aged more than 55 years and 2.2% (24/1089) aged 18 years and below. Among the five provinces of the study area, 44.9% (489/1089) of the participants were from Central Province, while 16.2% (176/1089) were from Darfur Province, 13.9% (151/1089) were from Merawe Province, Blue Nile Province and Kassala Province were the residences of 12.9% (141/1089) and 12.1% (132/1089) of the participants respectively. Concerning the educational level of the participants, 46.5% (506/1089) of them were college graduates, while 42.1% (458/1089) had either a primary or secondary level of education. On the other hand, participants who were illiterate or had Khalwa education comprised 11.5% (125/1089) of the study population. 1077 of the participants had reported if they were in the medical field or not. Of those, 76.9% (828/1077) were not in the medical field, and the remaining 23.1% (249/1077) were in the medical field. The marital status of the participants was assessed, 39.5% (425/1075) were never married. Of the participants, the group who were married in a consanguineous marriage or non-consanguineous marriage were 30.2% (325/1075) each (Table 1).

**Table 1:** Demographic characteristics of the study participants.

Characteristics	*n* (%)	Characteristics	*n* (%)
Age in years		Educational level (*n*=1089)	
18–35	701 (64.4)	Graduates/postgraduate degrees	506 (46.5)
36–55	310 (28.5)	Primary/high school graduates	458 (42.1)
> 55	78 (7.2)	Illiterate and khalwa	125 (11.5)
Sex (*n*=1089)		Participants in the medical field (*n*=1077)	
Male	518 (47.6)	No	828 (76.9)
Female	571 (52.4)	Yes	249 (23.1)
Residence (*n*=1089)		Marital status (*n*=1075)	
Central Province	489 (44.9)	Never married	425 (39.5)
Darfur Province	176 (16.2)	Married in CM^a^	325 (30.2)
Merawe Province	151 (13.9)	Married in NCM^b^	325 (30.2)
Blue Nile Province	141 (12.9)		
Kassala Province	132 (12.1)		

^a^ CM, consanguineous marriage.

^b^ NCM, non-consanguineous marriage.

### Assessment of the status of knowledge of participants about the health troubles associated with Consanguineous Marriage

The knowledge of participants about consanguineous marriage was assessed, they were asked if they were aware that health issues and inherited diseases can be related to consanguineous marriage. On assessing attitude and practice, participants were asked if they prefer consanguineous marriage or not and whether they advise it.

#### knowledge, attitude and practice of the study participants regarding Consanguineous Marriage

Almost two-thirds of the participants, 73.7% (803/1089), knew that consanguineous marriage may cause health troubles in offspring through hereditary factors, while 13.8% (150/1089) did not know that there was such a relationship. 12.5% (136/1089) who did not know about this. 63.9% (696/1089) of the participants did not prefer consanguineous marriage, while 36.1% (393/1089) preferred such practice. However, 44.3% (482/1089) of the participants had advised against consanguineous marriage, and 32.0% (394/1089) had advised for it (Table 2).

**Table 2:** Knowledge, attitude, and practice of the study participants regarding consanguineous marriage.

Knowledge, attitude and practice	*n* (%)
Are health troubles and inherited diseases related to consanguineous marriage? (*n*=1089)	
Yes	803 (73.7)
No	150 (13.8)
I don’t know	136 (12.5)
Is your spouse related to you by blood? (*n*=644)	
Yes	325 (48.9)
No	325 (48.9)
Do you prefer a consanguineous marriage? (*n*=1089)	
No	696 (63.9)
Yes	393 (36.1)
Do you advise your community about consanguineous marriage? (*n*=1089)	
No	482 (44.3)
Yes	349 (32.0)
Neutral	258 (23.7)

#### Association between knowledge and residence of participants

Correlation testing revealed a significant association between the level of knowledge and the residence of participants (*P*=0.000). (Table 3).

**Table 3:** Association between knowledge and residence of participants.

	Knowledge about health troubles of consanguineous marriage			
Residence	Yes, *n* (%)	No, *n* (%)	Total, *n* (%)	*p*
Blue Nile Province	105 (82.7)	22 (17.3)	127 (13.3)	0.000
Central Province	376 (87.0)	56 (13.0)	432 (45.3)	
Darfur Province	127 (80.9)	30 (19.1)	157 (16.5)	
Kassala Province	113 (91.9)	10 (8.1)	123 (12.9)	
Merawe Province	82 (71.9)	32 (28.1)	114 (12.0)	
Total	803 (84.3)	150 (15.7)	953 (100.0)	

#### Association between knowledge and characteristics of participants

Furthermore, correlation testing revealed a significant association between the level of knowledge and sex of participants (*P*=0.000), age of participants (*P*=0.035), marital status (*P*=0.000), educational level (*P*=0.000), preference of family marriage (*P*=0.000), and willingness to undergo premarital genetic testing (*P*=0.000). (Table 4).

**Table 4:** Association between knowledge and characteristics of participants.

	Knowledge about health troubles of consanguineous marriage				
Characteristics	Yes, *n* (%)	No, *n* (%)	Total, *n* (%)	*p*
Sex				
Male	353 (79.7)	90 (20.3)	443 (46.5)	0.000
Female	450 (88.2)	60 (11.8)	510 (53.5)	
Total	803 (84.3)	150 (15.7)	953 (100.0)	
Age				
≤18 years	15 (75.0)	5 (25.0)	20 (2.1)	0.035
19–35	511 (86.9)	77 (13.1)	588 (61.7)	
36–55	217 (80.7)	52 (19.3)	269 (28.2)	
> 55	60 (78.9)	16 (21.1)	76 (8.0)	
Total	803 (84.3)	150 (15.7)	953 (100.0)	
Marital status				
Never married	323 (87.8)	45 (12.2)	368 (38.6)	0.000
Married in CM	210 (74.7)	71 (25.3)	281 (29.5)	
Married in NCM	256 (88.3)	34 (11.7)	290 (30.4)	
Total	789 (84.0)	150 (16.0)	939 (98.5)	
The educational level of the participants				
Illitrate/khalwa	63 (66.3)	32 (33.7)	95 (10.0)	0.000
Primary/High school	335 (82.3)	72 (17.7)	407 (42.7)	
Graduates and postgraduates	405 (89.8)	46 (10.2)	451 (47.3)	
Total	803 (84.3)	150 (15.7)	953 (100.0)	
Do you prefer a consanguineous marriage?				
Yes	231 (72.4)	88 (27.6)	319 (33.5)	0.000
No	572 (90.2)	62 (9.8)	634 (66.5)	
Total	803 (84.3)	150 (15.7)	953 (100.0)	
Are you willing to undergo premarital genetic testing?				
Yes	602 (85.8)	100 (14.2)	702 (73.7)	0.000
No	101 (72.7)	38 (27.3)	139 (14.6)	
Total	803 (84.3)	150 (15.7)	953 (100.0)	

CM, consanguineous marriage; NCM, non-consanguineous marriage.

### Assessment of preference of participants of consanguineous marriage

#### Reasons reported by the participants encouraging consanguineous marriage

**Figure 1:**
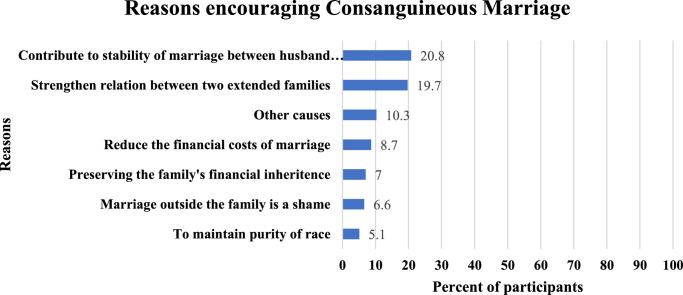
Reasons reported by the study participants encouraging consanguineous marriage. (*n*=1089).

Of all 1089 participants, 20.8% encouraged consanguineous marriage because it contributes to the stability of marriage between husband and wife. The second prevalent reason for encouraging consanguineous marriage was strengthening relationships between two extended families. Other reasons are detailed in Fig. 1.

#### Reasons reported by the participants encouraging consanguineous marriage

**Figure 2:**
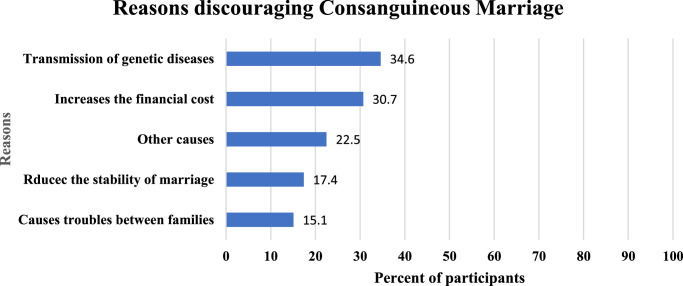
Reasons reported by the study participants discouraging consanguineous marriage. (*n*=1089).

The most prevalent reasons discouraging consanguineous marriage were the transmission of genetic disease and increases in the financial costs reported by 34.6% and 30.7% of the participants, respectively. Other reasons discouraging consanguineous marriage are detailed in Fig. 2.

#### Association between characteristics of the participants and their preference for consanguineous marriage

The study assessed the association between the characteristics of the participants and their preference for consanguineous marriage. On assessing age, younger participants who were aged in the range between 18 or less and 19 years to 35 years did not prefer consanguineous marriage (70.8% of each group of those participants). Older participants who did not prefer consanguineous marriage were 53.9% (167/310) of the age group 36–55 years and 42.3% (33/78) of the participants aged above 55 years. A statistically significant association (*P*=0.000) was found between age and preference for consanguineous marriage, table 5. Regarding sex, 518 participants were males, out of which, 57.5% (289/518) did not prefer consanguineous marriage. On the other hand, among the 571 females, 69.7% (398/571) did not prefer consanguineous marriage. There was a statistically significant association (*P*=0.000) between sex and preference for consanguineous marriage.

Similarly, a strong association was found between preference for consanguineous marriage and educational level, the highest percentage of participants who preferred consanguineous marriage were the illiterate and Khalwa group (60.8%, 76/125), followed by the group of primary and secondary education (42.4%, 194/458). The group of participants who were highly educated (university graduates and postgraduates) had the lowest preference for consanguineous marriage (24.3%, 123/506), and a statistically significant association was found (*P*=0.000) between the preference for consanguineous marriage and educational level of the participants. Participants with medical background had lower preference than the group of participants with no medical background (26.5%, 66/249 and 39.1, 324/828, respectively), there was a statistically significant association found (*P*=0.000) between having a medical background and preference of consanguineous marriage. Participants whose spouses were related to them by blood had a high preference for consanguineous marriage (68%, 221/325), in the other hand, participants who were not related to their spouses had a low preference for consanguineous marriage (22.2%, 72/325). A statistically significant association was found (*P*=0.000) between the preference for consanguineous marriage and blood relationship, (Table 5).

**Table 5:** Association between characteristics of the participants and their preference for consanguineous marriage.

	Preference for consanguineous marriage				
Characteristics	Yes, *n* (%)	No, *n* (%)	Total, *n* (%)	χ^2^	*p*
Age in years					
≤18 years	7 (29.2)	17 (70.8)	24 (2.2)	43.6	0.000
19–35	198 (29.2)	479 (70.8)	677 (62.2)		
36–55	143 (46.1)	167 (53.9)	310 (28.5)		
> 55	45 (57.7)	33 (42.3)	78 (7.2)		
Total	393 (36.1)	696 (63.9)	1089 (100.0)		
Sex					
Male	220 (42.5)	298 (57.5)	518 (47.6)	17.5	0.000
Female	173 (30.3)	398 (69.7)	571 (52.4)		
Total	393 (36.1)	696 (63.9)	1089 (100.0)		
Marital status					
Never married	98 (23.1)	327 (76.9)	425 (39.5)	201.4	0.000
Married in CM	221 (68.0)	104 (32.0)	325 (30.2)		
Married in NCM	72 (22.2)	253 (77.8)	325 (30.2)		
Total	391 (36.4)	684 (63.6)	1075 (100.0)		
Educational level					
Illitrate and khalwa	76 (60.8)	49 (39.2)	125 (11.5)	71.3	0.000
Primary and high school graduates	194 (42.4)	264 (57.6)	458 (42.1)		
Graduates and postgraduate degrees	123 (24.3)	383 (75.7)	506 (46.5)		
Total	393 (36.1)	696 (63.9)	1089 (100.0)		
Participants with a medical background					
Yes	66 (26.5)	183 (73.5)	249 (23.1)	13.2	0.000
No	324 (39.1)	504 (60.9)	828 (76.9)		
Total	390 (36.2)	687 (63.8)	1077 (100.0)		
Is your spouse related to you by blood?					
I prefer not to say	2 (14.3)	12 (85.7)	14 (2.1)	143.6	0.000
Yes	221 (68.0)	104 (32.0)	325 (48.9)		
No	72 (22.2)	253 (77.8)	325 (48.9)		
Total	295 (44.4)	369 (55.6)	664 (100.0)		

CM, consanguineous marriage; NCM, non-consanguineous marriage.

#### Association between the residence of the participants and their preference for consanguineous marriage

Correlation testing revealed a significant association between preference for Consanguineous Marriage and residence of participants (*P*=0.000). (Table 6).

**Table 6:** Association between the residence of the participants and their preference for consanguineous marriage

	Preference for consanguineous marriage			
Residence	Yes, *n* (%)	No, *n* (%)	Total, *n* (%)	*p*
Blue Nile Province	36 (25.5)	105 (74.5)	141 (12.9)	0.000
Central Province	144 (29.4)	345 (70.6)	489 (44.9)	
Darfur Province	101 (57.4)	75 (42.6)	176 (16.2)	
Kassala Province	29 (22.0)	103 (78.0)	132 (12.1)	
Merawe Province	83 (55.0)	68 (45.0)	151 (13.9)	
Total	393 (36.1)	696 (63.9)	1089 (100.0)	

### Assessment of attitude and practice of the study participants regarding premarital genetic testing

Participants were asked about their attitudes towards premarital genetic testing and their willingness to undergo premarital genetic testing. The practice of the participants was assessed by asking them if they were willing to undergo consanguineous marriage despite the unfavourable results of genetic testing.

#### Attitude and practice of the study participants regarding premarital genetic testing

Of the 1089 participants, 71.5% (7798/1089) were willing to undergo genetic testing before marriage, only 17.4% (190/1089) were unwilling to do so, and 11.0% (120/1089) did not know. Regarding marriage despite the unfavourable test results, 52.9% (576/1089) were not willing to get married, on the other hand, 30.5% (332/1089) of the participants were still willing to proceed with marriage despite the unfavourable results of the genetic tests (Table 7).

**Table 7:** Attitude and practice of the study participants regarding premarital genetic testing (*n*=1089).

Attitude and practice	*n* (%)
Are you willing to undergo premarital genetic testing?	
Yes	779 (71.5)
No	190 (17.4)
Do not know	120 (11.0)
Marriage despite the unfavourable results of genetic testing	
No	576 (52.9)
Yes	332 (30.5)
Do not know	181 (16.6)

#### Association between residence and willingness to get premarital genetic testing

Correlation testing revealed a significant association between the willingness to conduct premarital genetic testing and the residence of participants (*P*=0.000). (Table 8).

**Table 8:** Association between residence and willingness to undergo genetic testing

	Willingness to undergo premarital genetic testing			
Residence	Yes, *n* (%)	No, *n* (%)	Total, *n* (%)	*p*
Blue Nile Province	97 (70.3)	41 (29.7)	138 (14.2)	0.000
Central Province	416 (85.8)	69 (14.2)	485 (50.1)	
Darfur Province	142 (80.7)	34 (19.3)	176 (18.2)	
Kassala Province	19 (100.0)	0	19 (2.0)	
Merawe Province	105 (69.5)	46 (30.5)	151 (15.6)	
Total	779 (80.4)	190 (19.6)	969 (100.0)	

#### Reasons reported by the participants for unwilling to take premarital genetic testing

**Figure 3:**
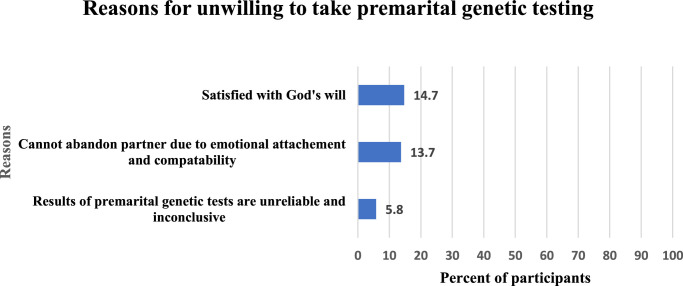
Reasons reported by the study participants for unwilling to take premarital genetic testing (*n*=190).

The 190 participants who were unwilling to undergo genetic testing were asked to report their reasons for being unwilling to take premarital genetic testing, the most frequent reason reported was the satisfaction of the participants with God’s will (14.7%, 28/190), followed by cannot abandon their partners due to emotional attachment (13.7%, 26/190). 5.8% (11/190) of the participants reported that they consider genetic tests unreliable. Figure 3 illustrates the details.

#### Association between characteristics of the participants and their willingness to undergo premarital genetic testing

There was a statistically significant association between willingness to undergo premarital genetic testing and age (*P*=0.037), with the older participants being more willing to undergo premarital genetic testing, table 9. of the 571 female participants, 72.9% (416/571) were willing to undergo premarital genetic testing, and 13.8% (79/571) were not willing. Among the 518 male participants, 70.1% (3363/518) were willing to undergo premarital genetic testing, and 21.4% (111/518) were unwilling to undergo premarital genetic testing, a statistically significant association was found (*P*=0.001) between sex and willingness to undergo genetic testing. Similarly, a statistically significant association was found (*P*=0.001) between marital status and willingness to undergo premarital genetic testing.

Among the 125 illiterate participants, 53.6% (67/125) were willing to undergo premarital genetic testing, while 15.2% (19/125) were unwilling. Of those 458 who have primary and secondary education, 67% (307/458) were willing to undergo premarital genetic testing, and 18.6% (85/458) were unwilling. Participants with a university level of education were the most willing group to undergo premarital genetic testing (80%, 405/458). A statistically significant association was found (*P*=0.000) between educational level and willingness to undergo premarital genetic testing.

Participants with medical backgrounds were 249 participants. Of those, 66.7% were willing to undergo premarital genetic testing. On the other hand, of the 828 participants with no medical background, 73.3% (607/828) were willing to undergo premarital genetic testing (*P*=0.046), (Table 9).

**Table 9:** Association between characteristics of the participants and their willingness to undergo premarital genetic testing.

	Willingness to undergo premarital genetic testing					
	Don’t know, *n* (%)	Yes, *n* (%)	No, *n* (%)	Total, *n* (%)	χ^2^	*p*
Age in years						
≤18 years	3 (12.5)	16 (66.7)	5 (20.8)	24 (2.2)	13.4^c^	0.037
19–35	74 (10.9)	466 (68.8)	137 (20.2)	677 (62.2)		
36–55	36 (11.6)	239 (77.1)	35 (11.3)	310 (28.5)		
> 55	7 (9.0)	58 (74.4)	13 (16.7)	78 (7.2)		
Total	120 (11.0)	779 (71.5)	190 (17.4)	1089 (100.0)		
Sex						
Male	44 (8.5)	363 (70.1)	111 (21.4)	518 (47.6)	15	0.001
Female	76 (13.3)	416 (72.9)	79 (13.8)	571 (52.4)		
Total	120 (11.0)	779 (71.5)	190 (17.4)	1089 (100.0)		
Marital status						
Never married	62 (14.6)	272 (64.0)	91 (21.4)	425 (39.5)	21.3^c^	0.001
Married in CM^a^	26 (8.0)	247 (76.0)	52 (16.0)	325 (30.2)		
Married in NCM^b^	29 (8.9)	251 (77.2)	45 (13.8)	325 (30.2)		
Total	117 (10.9)	770 (71.6)	188 (17.5)	1075 (100.0)		
Educational level						
Illitrate and khalwa	39 (31.2)	67 (53.6)	19 (15.2)	125 (11.5)	95.6	0.000
Primary/high school graduates	66 (14.4)	307 (67.0)	85 (18.6)	458 (42.1)		
Graduates/postgraduates	15 (3.0)	405 (80.0)	86 (17.0)	506 (46.5)		
Total	120 (11.0)	779 (71.5)	190 (17.4)	1089 (100.0)		
Participants with a medical background						
Yes	37 (14.9)	166 (66.7)	46 (18.5)	249 (22.9)	6.2	0.046
No	80 (9.7)	607 (73.3)	141 (17.0)	828 (76.0)		
Total	117 (10.9)	773 (71.8)	187 (17.4)	1077 (98.9)		
Is your spouse related to you by blood?						
I prefer not to say	3 (21.4)	9 (64.3)	2 (14.3)	14 (1.3)	2.9^c^	0.578
Yes	26 (8.0)	247 (76.0)	52 (16.0)	325 (29.8)		
No	29 (8.9)	251 (77.2)	45 (13.8)	325 (29.8)		
Total	58 (8.7)	507 (76.4)	99 (14.9)	664 (61.0)		

a CM, consanguineous marriage.

b NCM, non-consanguineous marriage.

c Likelihood ratio.

### Factors predicting knowledge, preference for consanguineous marriage and willingness to undergo premarital genetic testing among participants

Logistic regression models were developed to predict the Factors predicting knowledge, preference of consanguineous marriage and willingness to undergo premarital genetic testing among participants through using different factors.

Factors predicting knowledge about health troubles of consanguineous marriage among participants (Table 10).

**Table 10:** Logistic regression for factors predicting knowledge about health troubles of consanguineous marriage among participants.

							95% CI for OR	
Knowledge	B	Std. Error	Wald	df	*p*	OR	Lower	Upper
Intercept	0.168	1	0.028	1	0.867			
Age	0.005	0.155	0.001	1	0.976	1.005	0.742	1.361
Sex	0.359	0.209	2.953	1	0.086	1.431	0.951	2.155
The educational level of the participants	0.547	0.161	11.494	1	0.001	1.729	1.26	2.372
Is your study\job within the medical field?	−0.35	0.275	1.617	1	0.204	0.705	0.411	1.209
Marital status	−0.022	0.146	0.023	1	0.879	0.978	0.734	1.302
Transmission of genetic diseases	1.087	0.316	11.862	1	0.001	2.967	1.598	5.508
Premarital genetic testing	−0.797	0.24	10.994	1	0.001	0.451	0.282	0.722
Do you prefer a consanguineous marriage?	0.633	0.239	7.033	1	0.008	1.882	1.179	3.004

OR, odds ratio.

### Factors predicting preference for consanguineous marriage among participants

Our equation for the regression model fitted perfectly the model (*P*=0.000). The equation was as follows:

Preference of consanguineous marriage = −1.464 + (0.372*Age) + (−0.477* Sex) + (−0.118*Marital status) + (−0.6*Educational level) + (0.38* Participants in the medical field) + (4.971*Contribute to stability of marriage) + (5.37* Strengthen relationship between families).

The model revealed that Contributing to the stability of marriage and strengthening the relationship between families were the factors of high statistical significance (*P*=0.000), followed by the educational level of the participants (*P*=0.001) and sex (*P*= 0.047), (Table 11).

**Table 11:** Logistic regression for factors affecting the preference of participants for consanguineous marriage.

							95% CI for OR	
Factors	B	SE	Wald	df	*p*	OR	Lower	Upper
Intercept	−1.464	0.968	2.287	1	0.13			
Age	0.372	0.193	3.727	1	0.054	1.451	0.994	2.117
Sex	−0.477	0.24	3.947	1	0.047*	0.621	0.388	0.994
Marital status	−0.118	0.15	0.62	1	0.431	0.889	0.662	1.192
Educational level	−0.6	0.177	11.516	1	0.001*	0.549	0.388	0.776
Participants in the medical field	0.38	0.304	1.562	1	0.211	1.462	0.806	2.654
Contribute to the stability of marriage	4.971	0.415	143.532	1	0.000*	144.24	63.95	325.3
Strengthen relationships between families	5.37	0.534	100.998	1	0.000*	214.78	75.37	612.1

OR, odds ratio.

* Statistically significant.

### Factors predicting willingness to undergo premarital genetic testing among participants

Our equation for the regression model perfectly fitted the model (*P*=0.000). The equation was as follows: willingness to undergo premarital genetic testing = 0.131+ (0.279*Age) + (0.664* Sex) + (0.125*Educational level) + (0.706* Participants in the medical field) + (-0.348* Marital status) (0.178* Participants and their spouses are blood-related) + (-0.801* Health troubles and inherited diseases related to) + (0.337* Transmission of genetic diseases). (Table 12).

**Table 12:** Logistic regression for factors affecting willingness of participants to undergo premarital genetic testing.

							95% CI of OR	
Factors	B	SE	Wald	df	*p* value	OR	Lower	Upper
Intercept	0.131	1.157	0.013	1	0.910			
Age in years	0.279	0.184	2.297	1	0.130	1.322	0.922	1.895
Sex	0.664	0.243	7.46	1	0.006*	1.943	1.206	3.129
Educational level	0.125	0.18	0.483	1	0.487	1.133	0.797	1.611
Participants in the medical field	0.706	0.283	6.235	1	0.013*	2.025	1.164	3.524
Marital status	-0.348	0.327	1.133	1	0.287	0.706	0.372	1.34
Participants and their spouses are blood-related	0.178	0.314	0.321	1	0.571	1.195	0.646	2.21
Health troubles and inherited diseases related to consanguineous marriage	-0.801	0.155	26.883	1	0.000*	0.449	0.331	0.608
Transmission of genetic diseases	0.337	0.298	1.276	1	0.259	1.4	0.781	2.51

OR, odds ratio.

* Statistically significant.

## Discussion

The Messenger of Allah said: “Marriage is part of my sunnah, and whoever does not follow my sunnah has nothing to do with me. Get married, for I will boast of your great numbers before the nations. Whoever has the means, let him get married, and whoever does not, then he should fast for it will diminish his desire.”

The persistence of consanguineous marriages remains noteworthy in the face of significant social and demographic changes worldwide. Despite these changes, our study sheds light on the continuing prevalence of consanguinity, especially in Sudan, where 52% of our married respondents reported being married to a relative. This finding aligns with previous reports of high consanguinity rates in Sudan^10^.

Literature indicates that within different ethnic and religious communities, there exists a preference for consanguineous marriages, although the extent of this preference varies. Moreover, it is evident that parents remain the primary decision-makers regarding the marriages of both their sons and daughters. The main motivations behind this preference are predominantly socio-cultural rather than driven by perceived economic advantages, such as the consolidation of family assets or the reduction of dowry expenses. Notably, among Muslims, adherence to religious customs ranks lowest among the stated reasons for opting for consanguineous marriages. Despite the perceived socio-cultural benefits associated with such unions, there is a prevalent perception that they perpetuate existing power dynamics within families, leading to their characterization as exploitative^31^. This manifested in the findings of a study conducted in Jordan in 2018, where the prevalence of family marriage was found to be 27.5%, while the prevalences of divorced/separated rates was 4.7%. Compared to non-consanguineous marriages, the risk of divorce/separation was found to be lower among women with consanguineous marriages, while the survival of marriages was found to be higher for consanguineous marriages than for non-consanguineous marriages^32^.

Our study uncovered a nuanced situation in Sudan, with respondents who condemned consanguinity as a cause of hereditary diseases while simultaneously indicating a high level of preference for consanguineous marriage. This apparent contradiction prompted our investigation into the knowledge, attitudes, and practices of the population. Examining the knowledge of the ill-health consequences of consanguineous marriage, we found a substantial level of awareness (73.7%) among our respondents regarding the potential impact of intra-familial marrying on offspring health. This awareness level is comparable to that reported in the Omani population (69%) but appears notably higher than observations in India and the Netherlands^33,34^.

Interestingly, our finding contradicted our initial hypothesis that increased awareness of potential adversities would discourage the practice of marrying relatives. We anticipated that a highly consanguineous society might be inclined to reject this information. However, the majority of respondents who were both aware of the health impacts of consanguineous marriage and preferred such unions accounted for (72.4%). Furthermore, among those without knowledge yet expressing a preference for consanguineous marriage, the percentage was notably high at (90.2%).

To delve deeper into this apparent discordance, we compared the level of knowledge about health impacts with the preference for consanguinity. Contrary to expectations, respondents who were aware of the health impacts were not necessarily more likely to have a negative attitude toward consanguinity. Additionally, those who were aware but still preferred consanguineous marriage constituted a significant proportion (72.4%). The results challenged the assumption that knowledge alone would deter the practice of consanguinity in our society. Further analysis revealed that never-married individuals (87.8%) and those married in non-consanguineous unions (88.3%) had slightly higher knowledge levels than those married to relatives (74.7%). These findings undermine the straightforward correlation between awareness and the avoidance of consanguineous marriage, emphasizing the need to consider other influential factors contributing to the continuity of this practice in our society.

In conclusion, while our study highlighted a significant level of awareness regarding the health implications of consanguineous marriage, the coexistence of this awareness with a high preference for such unions challenges conventional expectations. The complexity of factors influencing the persistence of consanguinity in our society requires further exploration, considering social, cultural, and possibly deeply rooted influences that contribute to the maintenance of this practice despite knowledge of its potential adverse effects.

It is noticeable in other studies that participants from uneducated backgrounds and who are older or considered to be from rural areas generally show the lowest levels of knowledge^33^. However, the bivariate analysis in Table 5 shows that different socio-economic and demographic factors did not have a significant differential effect on knowledge in our population. Rather, concerning education, all of our groups reported higher-than-expected awareness. The range in the groups of formal education was (from 82 to 90%). Although the untaught group of illiterates and Khalwa schoolers* had a lower level of knowledge (66%), it was considered to be moderately high for the uneducated part of our population. Education had a more unique association in the Omani population; where respondents with higher education were 16 times more likely to be aware of congenital effects of CM compared to illiterates^35^. It was there for thought that the practice persists due to the lack of awareness. But our results confine with the study of Koc *et al.* ^29^, where despite the marked urbanization and modernization in many inbreeding populations countries, consanguinity persists contrasting the role of education. While older respondents reported significantly less knowledge in many studies^34,35^ we found most of our age groups - including the elderly—to record a moderately high level of knowledge (Table 4).

Interestingly, more women than men in our respondents have indicated knowledge of the subject (10% in favour of females). Other reports were varying either conforming with our results^35^ or recording not much difference^35^. We noticed that sex was not highly differential regarding attitude towards consanguinity. (42.5%) out of our female respondents were preferring CM, and (30.3%) out of our male respondents were also preferring. It is difficult to consider an isolated effect of gender on the level of knowledge or preference. There are many variables to consider such as the educational opportunities for each sex and the representation of females and males in society. Reporting this variance is important in formulating future awareness programs.

Taking into consideration that Sudan is a multi-ethnic multi-religious country, although other studies found a wide disparity in the awareness across the regions of residence of respondents^35^, most of the areas in our study were rural and noted low-income inhabitants, except in central Sudan, which had an urbanized commodity with higher socio-economic status and high variety of ethnic composition due to incoming from other states for the better educational and job opportunities. Due to that, we expected the central province to score higher levels of knowledge, but our data shows high knowledge of participants in all 5 provinces (83–92% in all areas, although to a lesser extent in the Meroe province, 71.9%).

Our results underscore the need to measure the depth of genetic knowledge in the Sudanese community. We argue that genetic knowledge in Sudan is overall lacking, there, many factors could have affected the perceivably high levels of knowledge in our population. For instance, the absence of observable defects in all the children of consanguineous marriages could create confusion concerning the genetic origins of the disease. Moreover, the perception of this risk could also be affected by high mortality from other causes, specifically from infectious diseases, which are widespread in the tropics and subtropics where consanguinity is common^36^. We also owe this to the fact that informational demand is not high due to the general low expertise in genetic health issues. In Sudan, genetic science is first-time integrated in the final year of high school, and only for biology majors. According to all these factors, we assume that this high level of knowledge could be inspired by subjective rather than objective genetic knowledge, which might be coming from encounters of personal or societal experiences where complex family histories of consanguinity have led to miserable health outcomes.

The current attitudes towards CM could be used as a predictor of the future of this practice, so we investigated the preference of our population towards this trend. We found over half of our respondents (63.9%) to be non-preferring of consanguineous marriage and over the third (44.3%) to have clearly stated that they would advise their families and communities against it. Our population’s attitude had levelled out the reported in Oman, where (41.5%) indicated a negative attitude towards consanguineous marriage^35^. While in some consanguineous societies of the Western world, like Brazil, (79.3%) were against consanguineous marriage^37^. According to this comparison, we find that CM persists in many inbreeding populations despite the reported non-preference in many societies, including ours.

Factors such as illiteracy, low socio-economic status and old age are known to be connected to the preference for consanguinity^29^. When investigating these factors in our population, we found the elderly and illiterate respondents were more likely to have a positive attitude towards consanguineous marriage (54% and 60%, respectively).

These factors are also common in rural living, where consanguinity is highly linked to arranged marriages due to smaller marriage pools^16^. In such arranged unions, the will of the elderly in the family can delegate or even force couples to marry from extended families, where mostly the females are forced into these weddings at a young age. We notice in our study that more females than males express a non-favourable attitude (69.7%) vs. (57.5%), we raise this to the influence of compelling such unions on female partners. This observation further strengthens the role of awareness particularly among women.

The most prominent reason to prefer consanguineous marriage in our study was that intra-familial bonds support the stability of marriage (20.8%). The second most prevalent reason was to strengthen the relationship between the two extended families (19.7%). These factors came in accordance with what had been reported by Sandridge *et al*.^38^ in the Qatari population. We also found that those who have married a relative were two times more likely to have a positive attitude towards consanguineous marriage (68% of respondents), which is similar to reports from Oman^35^. Our findings show that preference for consanguinity can be an outcome of previous acquaintance. The precluded reflection on a consanguineous experience as a good experience with motivation to keep the family system intact supports the idea that consanguinity can perpetuate itself in extended families.

On the other hand, the most common motive against CM among those disfavouring consanguinity was the fear of transmitting genetic diseases. This is important as marriage outside the family was often perceived as a risky and disruptive option for health reasons by other studies^16^. The second most common cause was the financial costs, which was also striking because previous research has shown a preference for cousin marriage to coincide with lower costs of marriage reduced dowry and the preservation of landholdings within the family^39,40^.

A Sudanese study has put regard to social class in assortative mating, where in our community people tended to marry those of equal educational level^39^. In our study, we found an inverse relationship between the preference of CM and the level of education. Where (60.8%) were preferring of the illiterate and khalwa; (42.4%) of Primary and High school graduates, and (24.3%) of university and postgraduates. When comparing these results to the weak difference education has made in awareness in our population, we can there assume that the role of education in the non-preference of CM has more to do with social class and more societal exposure than with the depth of genetic knowledge.

In Table 10; multivariate logistic regression analysis was done to factors predicting the preference for consanguineous marriage among participants. We identified sex, levels of education, and contribution of intra-familial marriage to the stability of the relationship between spouses as significant positive associations.

Results of these regression tables indicate that a positive attitude towards consanguineous marriage remains a significant predictor of the current practice of consanguineous marriage after controlling other socio-economic and demographic factors, while awareness about congenital effects of consanguineous marriage shows no significant association with the practice of consanguineous marriage. We argue that this long method of marriage impeded in most of our population, along with genetic illiteracy, has led to putting more value on the continuation of this practice than its possible adverse effects on the health of the next generations.

Furthermore, we have investigated our population as future consumers of genetic services. We reported a very high acceptance of premarital genetic testing among the Sudanese population (83%). This corresponds to other studies where (70–80%) of respondents in consanguineous society have approved of DNA testing and found genetics positive in medical use^34^.

Moreover, we were interested in the attitude of people towards unions based on the outcomes of genetic tests; those willing to continue with the marriage despite unfavourable genetic test results were one-third of our respondents (30.5%). Among them, (13.4%) conveyed their choice to their absolute belief in fate. While (7.9%) did not want to abandon their partners due to emotional attachment. Only (3.1%) of the participants reported that they consider genetic tests unreliable.

The findings of this study show that religion is an important factor in this decision-making process. Submitting to God’s will, or what our respondents answered as fate, are beliefs influenced by their Islamic faith.

Interestingly, we found an equally high rate of acceptance in both sexes, which could contribute to conformed decision-making between couples (70.1% of males and 72.9% of females). Acceptance was also overall high when compared in terms of marital status; (76%) of those who are in the experience of consanguineous marriage, (77.2%) of non-consanguineous marriage, and (64%) of those who never married. No reserving attitude emerged concerning the experience of cousin marriage.

Although willingness to withstand GT was merely expected from those with higher education; we found that half of the illiterate participants (53.6%), were willing to undergo premarital genetic testing. Moreover, those having medical backgrounds were expected to have a more positive attitude towards genetics than those who weren’t in the medical field; however, only (66.7%) of those with medical backgrounds were willing to undergo premarital genetic testing. On the other hand, (73.3%) of those with no medical background were willing to undergo premarital genetic testing. This is striking because we expected the medical background to encourage participants to be familiar with the process and tests.

Many possible psychological and social factors could affect the convenience of genetic tests for people. When logistic regression was done, the model in Table 12 revealed that knowledge was the factor of highest statistical significance (*P*=0.000) in accepting genetic tests. People must be to some degree informed about genetics and genetic testing. Knowledge deficiency may lead people to refrain from taking a genetic test when necessary. Thus, it is quite likely that people’s beliefs about GT are positively affected when their genetic knowledge increases. It appears that although knowledge was not a discouraging factor towards CM when provided with precautious measures, our population had a great affinity to be prepared for possible outcomes.

## Conclusion

Consanguineous unions remain an integral part of the cultural and social life in Sudan. This research reports the discordance between the awareness of negative outcomes of consanguineous marriage and the high percentage of consanguineous marriages in our population. Any attempts to discourage consanguinity at the population level must regard the personal beliefs of the individuals. Our paper provides a wealth of evidence that the provision of public awareness programs about genetic risks, and thorough genetic counselling services in our consanguineous setting is highly necessary.

### Limitations

Despite the size of our population, the stratification of our population was limited by the difficulty of reaching some sub-populations due to geographical isolation and lack of funding. Occasional linguistic barriers have affected interviewing the depth of genetic knowledge. We report that in further studies, sub-populations must be approached in deeper stratification. In addition, it is recommended that further studies adopt up-to-date methods in data handling and analysis, such as *“text mining”* to draw specifically related articles^41^, *“social network mining”* ^42^, and *“data mining”* for relevant data bases^43^.

## Ethical approval

Ethical consent was obtained from the UNESCO Chair of Bioethics at the University of Khartoum, Khartoum, Sudan.

## Consent

Written informed consent was obtained from the patients for publication and any accompanying images. A copy of the written consent is available for review by the Editor-in-Chief of this journal on request.

## Source of funding

The study did not receive funding support.

## Author contribution

F.M.E., H.A., M.K.G., A.A. and I.A. wrote and drafted the first version of the manuscript and conceptualized it. A.A., M.S., W.M., E.A.A., K.A.H.M.A., G.E.M.A., L.E. and A.M. visualized, validated, conceptualized and wrote and drafted the manuscript’s final version and critically reviewed and edited the initial draft and proofread, and edited the manuscript’s final version. All authors reviewed and approved the final manuscript.

## Conflicts of interest disclosure

The authors declare no conflict of interest.

## Research registration unique identifying number (UIN)

1. Name of the registry: It is an observational study and the research registration was not required.
2. Unique Identifying number or registration ID: It is an observational study and the research registration was not required.
3. Hyperlink to your specific registration (must be publicly accessible and will be checked): It is an observational study and the research registration was not required.

## Guarantor

Khabab Abbasher Hussien Mohamed Ahmed.

## Data availability statement

The data that support the findings of this study are available from the corresponding author upon reasonable request.

## Provenance and peer review

Externally peer-reviewed, not commissioned.
